# Disseminated intravascular coagulation following air embolism during orthotropic liver transplantation: is this just a coincidence?

**DOI:** 10.1186/s12871-021-01476-6

**Published:** 2021-10-30

**Authors:** Karolina Arstikyte, Gintare Vitkute, Vilma Traskaite-Juskeviciene, Andrius Macas

**Affiliations:** 1 Medical Academy, Lithuanian University of Health Sciences, Kaunas, Lithuania; 2 Wakefield, UK; 3 Department of Anaesthesiology, Hospital of Lithuanian University of Health Sciences Kaunas Clinics, Kaunas, Lithuania

**Keywords:** Venous air embolism, Disseminated intravascular coagulation, Liver transplantation

## Abstract

**Background:**

During orthotopic liver transplantation, venous air embolism may occur due to iatrogenic injury of the inferior vena cava. However, venous air embolism followed by coagulopathy is a rare event. In this case report, we discuss a possible connection between venous air embolism and disseminated intravascular coagulation.

**Case presentation:**

A 37-year-old male patient with chronic hepatitis B- and C-induced liver cirrhosis was admitted for orthotopic liver transplantation. During the dissection phase of the surgery, arterial blood pressure, heart rate, saturation and end-tidal carbon dioxide levels suddenly decreased, indicating the occurrence of venous air embolism. After stabilizing the patient’s condition, various coagulation issues started developing. Venous air embolism-induced coagulopathy was handled by administering transfusions of various blood products. However, the patient’s condition continued to deteriorate leading to a complete asystole.

**Conclusions:**

This is a rare case of venous air embolism-induced disseminated intravascular coagulation. The real connection remains unclear as disseminated intravascular coagulation for end-stage liver disease patients can be induced by various causes during different stages of liver transplantation. Certainly, both venous air embolism and coagulopathy were significant and led to an unfavorable outcome. Further studies are needed to better understand the possible mechanisms and correlation between these two life-threatening complications.

## Background

The first human orthotopic liver transplantation (OLT) was performed in 1963. Despite the development of technology in the medical field, hepatic transplantation remains a difficult surgery and carries a risk of significant complications [1]. One possible, although rare, but potentially fatal complication that occurs during orthotopic liver transplantation is venous air embolism (VAE). It could be defined as the presence of air or carbon dioxide in the inferior vena cava and right atrium, which leads to the obstruction of blood flow through the heart [2]. Another well-known condition is coagulopathy, which is caused by liver disease and hemostatic changes during surgery [3] and can lead to the development of disseminated intravascular coagulopathy (DIC). However, we do not truly know if these two serious conditions are connected and whether one leads into another by any means. In other words, the prevalence of DIC following venous air embolism remains unclear. In this report, we describe how VAE was potentially followed by DIC during orthotopic liver transplantation, leading to a fatal outcome.

## Case presentation

A 37-year-old 70 kg male patient with chronic hepatitis B- and C-induced liver cirrhosis (MELD score of 28 and Child-Pugh of 11) presenting with mild jaundice and abdominal distension was admitted for orthotropic liver transplantation (OLT) to the Hospital of Lithuanian University of Health Sciences Kaunas Clinics. There was no clinical evidence of cardiopulmonary decompensation in physical examination or clinical history. CT CAP showed ground glass opacity (GCO) findings in the right lung. Arterial blood gas (ABG) values, electrocardiogram (ECG), spirometry, and transthoracic cardiac echo prior to surgery revealed no significant abnormalities. However, the coagulation profile was deranged: PT 43.6 s, APTT 60 s, fibrinogen 122 mg/dl, and platelets 36 × 10^9^/l. In the anticipation of a high risk of bleeding group and save as well as crossmatch were requested prior operation. In the operation theatre standard anesthesia was induced with 2.8 mcg/kg fentanyl, 2.5 mg/kg propofol, and 0.14 mg/kg cisatracurium. The patient was intubated with standard endotracheal tube no. 7.5 using direct laryngoscopy and MAC blade no.3, and ventilation was started using PRVC mode with Vt of 6–8 ml/kg IBW, 12 bpm, aiming for a moderately lower EtCO_2_ of 35 mmHg, a peak not exceeding 25 cm H_2_O and a positive end-expiratory pressure (PEEP) of 4 cm H_2_O. Sevoflurane (2.2–2.5% EtSEV) in an air-oxygen mixture, fentanyl on requirement and cisatracurium infusion were used for anesthesia maintenance. Central venous pressure, invasive arterial blood pressure and BIS monitoring were used together with all standard anesthesia monitoring. The ABG test was performed every hour. Rotational thromboelastometry (ROTEM) was used to guide blood product transfusion. According to scientific sources, ROTEM has proven to be more appropriate for the assessment of coagulation in liver diseases than conventional coagulation tests and it can reduce perioperative blood loss and blood product transfusion rates [4, 5]. Based on a local transplantation protocol, a rapid infusion system (The Belmont® Rapid Infuser (FMS 2000)) is routinely used in this transplantation center. Additionally, in anticipation of massive blood loss, the Hemonetics® Cell Saver Autologous Blood Recovery System is used unless contraindicated [6].

**Fig. 1:**
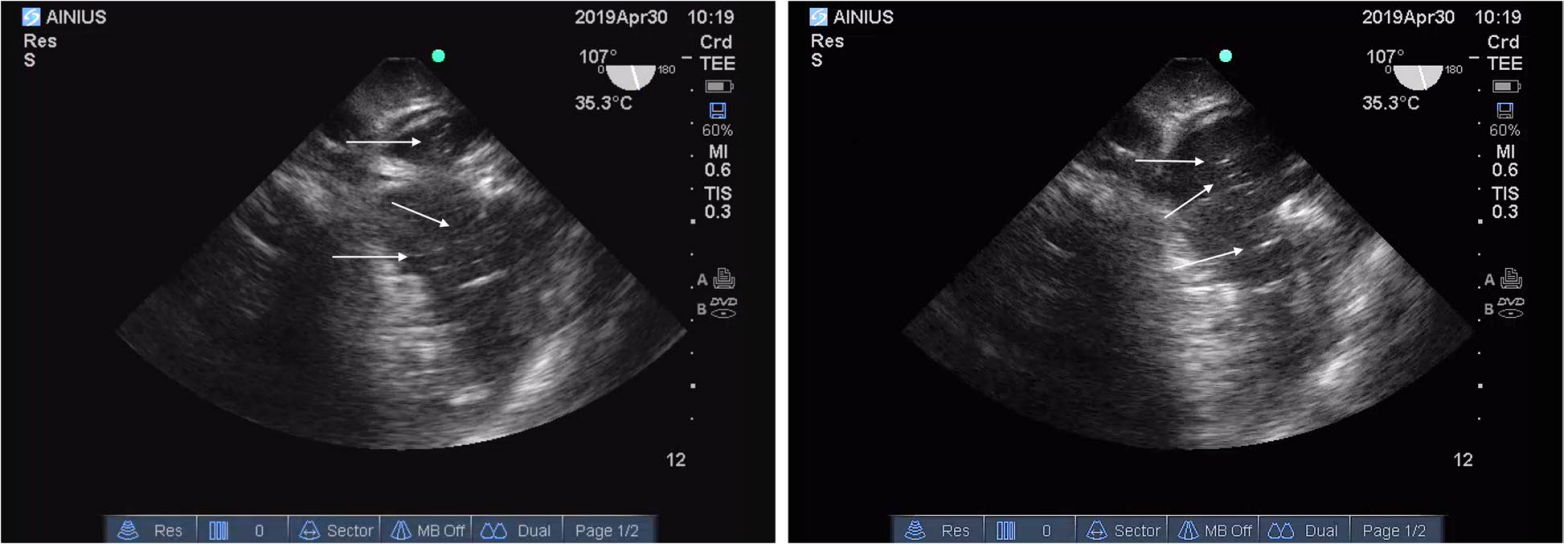
Arrows indicate air bubbles visible in both left and right cardiac chambers

**Fig. 2:**
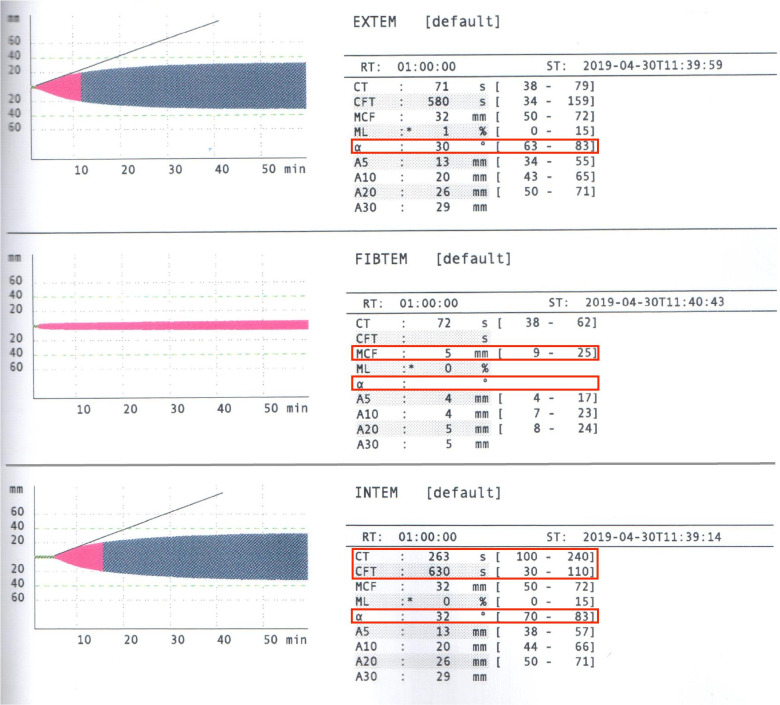
Poor levels of MCF in FIBTEM and dysfunctional intrinsic coagulation pathway (prolonged CT and CFT)) with sluggish alpha-angles in INTEM and EXTEM)

**Fig. 3:**
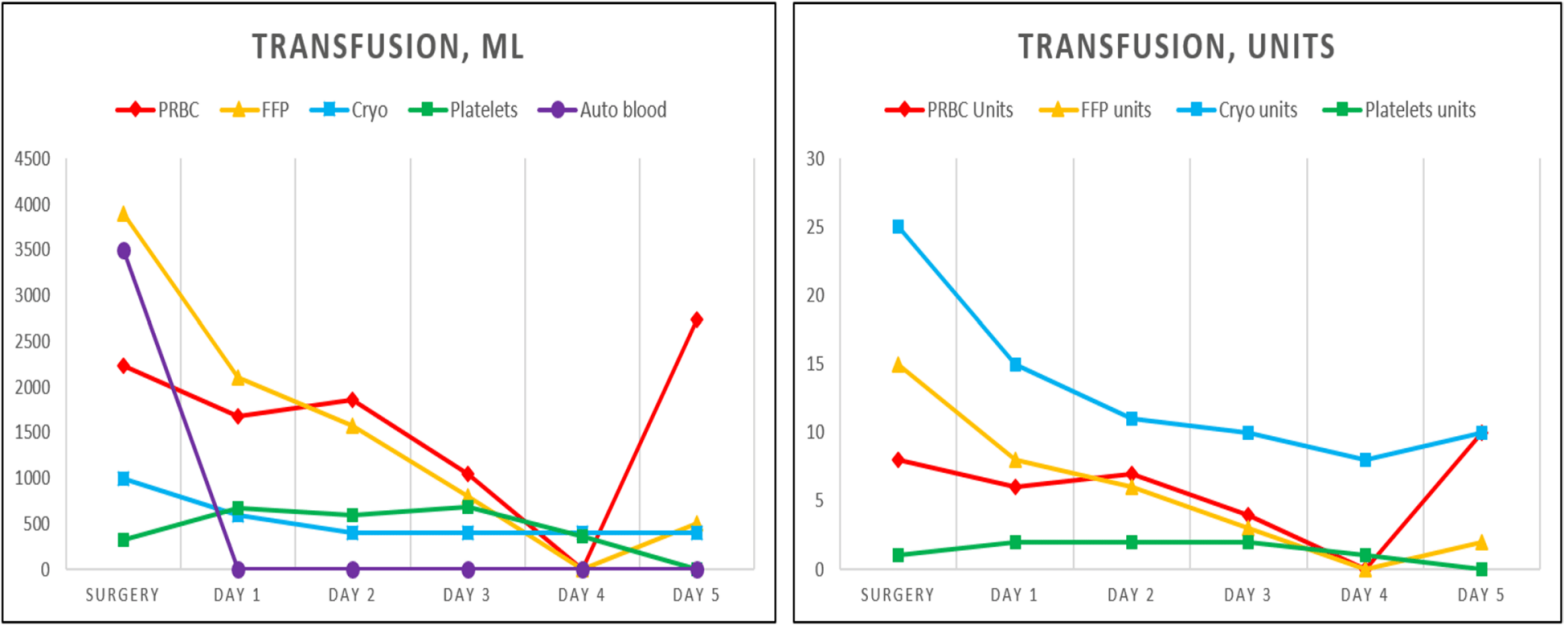
The dynamics of blood transfusion rates during the perioperative period

**Fig. 4:**
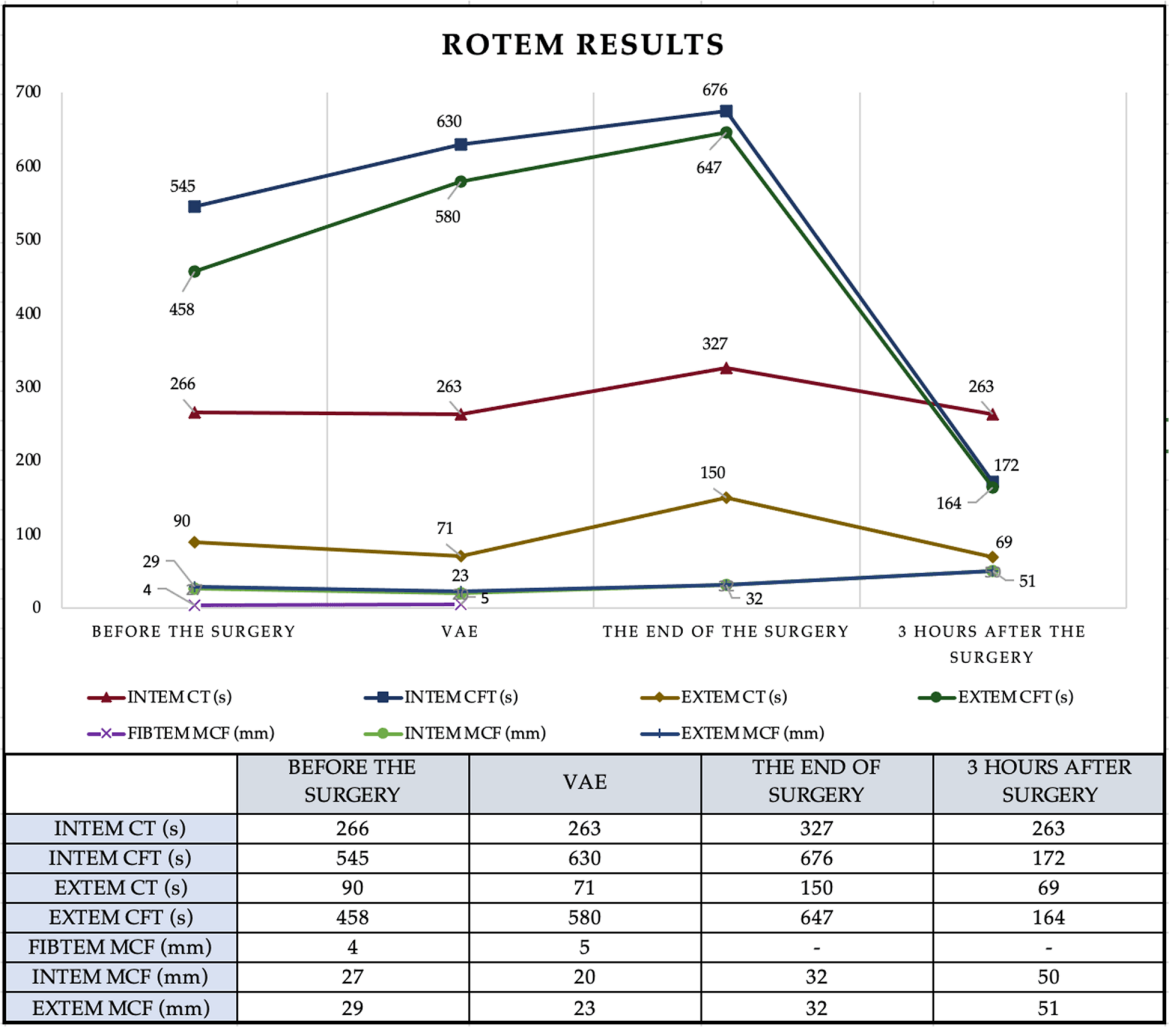
The dynamics of ROTEM on the transplantation day

There was no hemodynamic instability during the initial portion of the procedure. During the recipient hepatectomy (hour mark two), the patient had a sudden decrease in arterial blood pressure, heart rate, oxygen saturation and end-tidal carbon dioxide level. In high suspicion of air embolism, the oxygen fraction (FiO_2_) was instantly increased to 100% following immediate chest compressions and 1 mg of IV adrenaline. Mechanical ventilation was converted to manual ventilation to ensure adequate oxygenation. The resuscitation protocol was carried out for less than 2 min, proper asystole was never truly observed, and cardiovascular function was preserved. The surgeons confirmed the presence of a defect in the inferior vena cava at its junction with hepatic veins, proving the preliminary diagnosis of venous air embolism. After an unsuccessful attempt to aspirate air through the central venous catheter (CVC) and re-establishment of mechanical ventilation with a PEEP of 8 cm H_2_O, TEE was performed that revealed air bubbles in both the right and left heart chambers confirming the diagnosis (Fig. 1). Although adequate ventilation with a high inspired oxygen fraction was maintained, saturation remained low during the remaining portions of the surgical procedure. There were no additional episodes which represented VAE; however, further problems were more closely related to various coagulation issues which led to massive allogenic blood products and autologous blood transfusion and the development of DIC. Repeated viscoelastic coagulation tests revealed that CT, CFT in intrinsic and CFT in extrinsic coagulation pathways were severely prolonged due to significant deficiency of fibrinogen (MCF 5 mm). The urgent call of blood products was indicated – the total amount of transfused blood during the surgery was 10,955 ml (49 units) alongside 14,000 ml of fluids to maintain volemia. ROTEM and TEG both indicated severe coagulopathy with especially low fibrinogen levels (Fig. 2). The patient was transfused continuously using various blood products according to local transfusion protocols and viscoelastic blood coagulation test results (Fig. 3 and Table 1). The total amount of blood loss was 4000 ml. Intraoperative blood salvage (Cell Saver) was extremely beneficial as it allowed us to limit the amount of autologous blood transfusions and return 3500 ml of salvaged blood. Fibrinogen concentration was restored using cryoprecipitate, which may not have been as efficient or effective as compared to human fibrinogen concentrate, but was the only available agent at the time. Despite all interventions thrombocytopenia occurred (after VAE episode platelets decreased: 36- > 28 × 10^9^/l) due to the lack of fibrinogen, and proper clot formation could not be achieved (Fig. 4).

**Table 1:** The amount of substituted blood products and fluids after VAE-induced coagulopathy

		30/04–01/05 1st PO day		01/05–02/05 2nd day			02/05–03/05 3rd PO day	03/05–04/05 4th PO day	04/05–05-05 5th PO day	
		Tx (7 am-5 pm)	ICU (5 pm-7 am)	ICU (8 am-9 pm30)	Relaparotomy no. 1 (9 pm30-11 pm)	ICU 11 pm-7 am)	ICU (8 am–7 am)	ICU (8 am–4 am)	Relaparotomy no. 2 (4 am–6 am)	ICU (7 am-5 am25)
PRBC^a^ (ml)		2235 (8 U)	1675 (6 U)	521 (2 U)	800 (3 U)	540 (2 U)	1050 (4 U)	–	1339 (5 U)	1400 (5 U)
FFP^b^ (ml)	Rh+	3900 (15 U)	1831 (7 U)	781 (3 U)	795 (3 U)	–	799 (3 U)	–	510 (2 U)	–
	Rh-	–	279 (1 U)	–	–	–	–	–	–	–
Cryo^c^ (ml)	Rh+	1000 (25 U)	440 (11 U)	400 (10 U)	–	–	240 (6 U)	320 (8 U)	400 (10 U)	–
	Rh-	–	160 (4 U)	–	–	–	160 (4 U)	80 (2 U)	–	–
PLT^d^ (ml)		320 (1 U)	677 (2 U)	600 (2 U)	–	–	690 (2 U)	360 (1 U)	–	–
Auto-blood (ml)		3500	–	–	–	–	–	–	–	–
Total blood amount (ml)		10,955 (49 U)	5062 (31 U)	2302 (17 U)	1595 (6 U)	540 (2 U)	2939 (19 U)	760 (11 U)	2249 (17 U)	1400 (5 U)
		16,017 (80 U)		4437 (25 U)					2649 (22 U)	
Infusion (ml)		14,000	2000	2000	500	2500	1750	1500	1500	6500
		16,000		5000					8000	

^a^ -Packed red blood cells

^b^- Fresh frozen plasma

^c^- Cryoprecipitate

^d^- Platelets

U-units

Tx-transplantation

ICU-intensive care unit

PO day- post-operative day

During the other phases of the surgery, the patient received protective lung ventilation (Vt of 6–8 ml/kg IBW and PEEP at 8 cm H_2_O) with a high inspired oxygen fraction and 0.3 mcg/kg/min noradrenaline to maintain adequate ABP. The patient remained in a critical condition and was transferred to the ICU for further treatment. Repeated TTE revealed small ventricles with normal systolic function indicating consequences of hypovolemia secondary to severe bleeding.

Diffuse bleeding almost immediately after transplantation, on first postoperative day, from the upper abdomen and thrombosis of the hepatic artery were suspected as the patient’s anaemia worsened and transaminase levels increased. A computerized tomography (CT) scan demonstrated that the left hepatic artery was only partially filled with contrast leading us to suspect partial thrombosis as well as a large subhepatic heterogenic mass (6.5 cm × 7.5 cm) representing a possible hematoma. Contrast extravasation was also noted in the venous phase. The decision was made to perform emergency relaparotomy. Due to unstable hemodynamics caused by severe bleeding, coagulopathy and acidosis, noradrenaline was continuously given by reducing the dosage in a manner dependent on the requirement until complete cessation. Cryoprecipitate, prothrombin complex concentrate (PCC), platelets and packed red blood cells (PRBCs) were constantly given. Patient remained stable for 2 days before hypotension and anaemia worsened. The patient was taken back to the Operating Theatre for exploration which demonstrated 3000 ml of blood (fresh and clotted). Clotted blood was found suprahepatically around the caval anastomosis as well as porta hepatis. After the patient’s second laparotomy, he continued to deteriorate and required increasing doses of vasopressors to maintain adequate blood pressure and tissue perfusion. He continued to deteriorate becoming increasingly hypotensive, acidotic and eventually went into multisystem organ failure and died.

## Discussion and conclusions

Circulatory and metabolic problems associated with liver transplantation (LT) have been an issue since the beginning of these kinds of surgeries, despite improvements in surgical and anesthetic techniques [3]. The first stage of LT, when liver dissection is carried out, excessive bleeding can occur from collateral veins and arteriovenous malformations requiring massive transfusion of multiple blood products within a 24-h period [7, 8]. However, massive bleeding and transfusion increase the risk of transfusion-related acute lung injury (TRALI), allergic reactions, transfusion-related sepsis, hyper- or hypokalemia and other adverse reactions [9]. Even modest transfusion requirements have been linked with longer average lengths of hospital stay [8].

During the anhepatic phase, no hepatic clotting factors are produced, fibrinogen is depleted and antithrombin concentrations decrease, leading to worsening coagulopathy and the onset of fibrinolysis. Hyperfibrinolysis is detected in 30 to 46% of patients who have end-stage liver disease [2]. It may occur due to the reduced clearance of t-PA. Additionally, the reperfused graft releases t-PA and tissue factor, which may lead to DIC with fibrinolysis [7]. Likewise, depletion in fibrinogen, which is the major plasma protein coagulation factor and is vital for the proper coagulation cascade, is very prevalent. Despite the fact that fibrinogen levels in end-stage liver disease (ESLD) are typically normal or elevated, most of the proteins are dysfunctional due to abnormal molecular structure [10]. It is known that the fibrinogen range during surgery can decrease due to hemorrhage followed by infusions of fibrinogen-poor fluids or blood components, resulting in a vicious unstoppable bleeding cycle for ESLD patients [11]. Fibrinogen concentration can reduce the requirement of red blood cells, fresh frozen plasma and platelet transfusion by more than 50%, making this glycoprotein a desirable material in every operating room [12]. However, during the time this case occurred, fibrinogen was not officially registered in our country, making fibrinogen replacement therapy difficult or even impossible to administer. Fibrinogen was also not the first treatment of choice due to its several limitations, including high price and large volumes of the concentrate required to achieve a good therapeutic effect. According to viscoelastic coagulation test-guided patient blood management principles, aiming to maintain a FIBTEM MCF above 8 mm for patients who are bleeding or are likely to bleed would be a suitable approach that could also allow a decreased transfusion requirement [13]. Unfortunately, this benchmark failed to be achieved during the surgery (Fig. 3).

There is another possible explanation for abnormal coagulation in this case as the patient experienced cardiac arrest immediately after the episode of venous air embolism. Postcardiac arrest syndrome (PCAS) is often associated with DIC. The pathophysiology underlying systemic ischemia and reperfusion are coagulopathy and severe systemic inflammatory response. Impaired microcirculation might result in thrombotic vascular occlusion, which is known as DIC [14]. Coagulofibrinolytic changes during PCAS are characterized by tissue factor-dependent coagulation, which is accelerated by abnormal anticoagulant mechanisms, including antithrombin, protein C, thrombomodulin, and tissue factor pathway inhibitors. Damage-associated molecular patterns (DAMPs) initiate tissue factor-dependent coagulation and activate factor XII- and factor XI-dependent coagulation. In the early phase of PCAS, hyperfibrinolysis is followed by inadequate fibrinolysis and fibrinolytic shutdown [15].

Dilutional thrombocytopenia as an adverse effect of massive blood transfusion exacerbate preoperative thrombocytopenia in patients with chronic liver disease [16]. Additionally, it is important to mention an imbalance in pro- and anticoagulation factors in the plasma of cirrhotic patients, as the plasma of these patients’ is resistant to thrombomodulin, which is the main activator of the protein C anticoagulant pathway, resulting in hypercoagulability [17]. In hepatic insufficiency, anticoagulant antithrombin, protein C and protein S are reduced, as are procoagulant proteins, with the exception of factor VIII. Coagulation defects have been demonstrated pretransplantation, but hemostasis in most cases is rebalanced because of a deficit in pro- and anticoagulation factors [8]. The majority of clotting tests, such as PT and APTT, aim to measure procoagulant capacity and do not assess compensatory effects within the system, making them useless for estimating perioperative bleeding risk [18]. Point-of-care hemostatic tests such as viscoelastic tests (VETs), rotational thromboelastometry and thromboelastography provide more accurate assessments, making it possible to provide targeted controlled patient blood management [8]. VETs provide information about the kinetics of clot formation and the strength of the clot and distinguish contributions from fibrinogen, platelets and the fibrinolytic system [19].

The leading causes of air embolism are mostly mechanical defects caused by surgery, trauma, vascular interventions and barotrauma from mechanical ventilation or, rarely, diving [20]. Although air embolus is not an uncommon complication, there are only a few case reports of massive air embolus during orthotopic liver transplantation. The clinical features of VAE depend upon the rate and the volume of air entrained [21]. Acute complications such as VAE during surgery are often identified using TOE. This minimally invasive monitor is the most sensitive device for VAE and can detect 0.02 ml/kg air [22]. In our case, a defect in the hepatic vein and IVC confluence occurred during the dissection phase of the operation. Based upon TEE findings, studies have found that isolated right ventricular failure secondary to paradoxical emboli may result in hemodynamic instability during LT [23]. It embraces taking TEE into consideration not only for emergency situations such as VAE but also for intraoperative monitoring [24]. The opportunity to capture an image of discrepancies in heart chambers on cardiac echo throughout the surgery plays a crucial role in our case in confirming the diagnosis and dealing with VAE as early as possible.

VAE-induced intraoperative hemodynamic instability is first managed by maximizing the high oxygen fraction in inspired air [20]. A large analysis of pulmonary artery catheters (PACs) in high-risk patients during cardiac surgery failed to demonstrate an increase in morbidity and mortality and was associated with a longer length of stay in the intensive care unit and a longer duration of mechanical ventilation [25]. In our institution, PAC is used only for high-risk OLT patients considering the risk-benefit ratio despite the global recommendation not to use PAC routinely in patients who were considered to be at a high surgical risk, as further studies claim that it does not improve the outcome [26]. Moreover, PAC performs worse than other less invasive methods where the assessment of cardiac function and volume status is required. The prediction of fluid responsiveness is difficult, as assessing preload is not the same as assessing the response of preload [27].

Another possible explanation of coagulopathy or fibrinolysis, acidosis and hemodynamic instability is primary nonfunction (PNF) leading to retransplantation or death. PNF also causes hyperkalemia with oliguria or anuria, hypoglycemia and absence of bile, which were not present in our case [28]. Unfortunately, we could not obtain access to the characteristics of used liver graft.

DIC following air embolism during orthotopic liver transplantation is a rare case, and only a few studies have investigated the possible connection between these two complications. One study reported that VAE induces platelet dysfunction and thrombocytopenia [29]. A study with animals revealed that platelet aggregation and the release of plasminogen-activator inhibitors can be a result of the formation of microbubbles [30]. In our case, the reason for intraoperative coagulopathy might have been hypovolemia, although fluid resuscitation is limited in LT patients, especially during the dissection phase. Additionally, there was a similar case of VAE followed by impaired coagulation in 2013 when an 18-year-old female patient without any previous clinical history of chronic diseases and normal coagulation parameters underwent craniotomy and excision of a mid-brain ependymoma. Both our case and this case share the same cause of VAE, which was a surgical trauma in the venous system. However, female patient had two episodes of VAE: the first episode was hemodynamically insignificant (the decrease in ET carbon dioxide level was successfully treated with 100% oxygen), while the second episode was followed by hypotension and ST-T depression. Unfortunately, deterioration continued in both patients and they were declared dead [31]. The real and evidence-based connection between VAE and DIC remains unknown.

One of the contributing factors in our case is a lack of effective communication between the surgeon and the anesthetist. In 2013, a study conducted in India revealed that 52,2% of the surveyed anesthesiologists felt that poor communication between the surgeon and anesthesiologist affected the outcome [32]. Additionally, anesthesiologists emphasize the impact of surgeons being uneducated about anesthesia-related issues [33]. Only the acknowledgment of personal flaws, tactfulness and communication with colleagues can lead to safe, confident and patient-oriented teamwork in the operating room.

Although coagulopathy following venous air embolism has been reported in a small number of cases, it should not be underestimated, as some complications*,* although rare*,* may be life-threatening. The real connection remains unclear, although both problems were significant and led to an unfavorable patient outcome. Further studies are needed to better understand the possible mechanisms and correlation between these two life-threatening complications that occurred in this case.

## Data Availability

The datasets generated and analyzed during the current study are not publicly available due to preservation of the individual’s privacy under the European General Data Protection Regulation but are available from the corresponding author on reasonable request.

## Abbreviations

ABG: Arterial blood-gases
CVC: Central venous catheter
DAMPs: Damage-associated molecular patterns
DIC: Disseminated intravascular coagulopathy
ECG: Electrocardiogram
ESLD: End-stage liver disease
FFP: Fresh frozen plasma
FiO_2_: Oxygen fraction
GGO: Ground glass opacity
IVC: Inferior vena cava
LT: Liver transplantation
OLT: Orthotopic liver transplantation
PAC: Pulmonary artery catheters
PCAS: Post cardiac arrest syndrome
PCC: Prothrombin complex concentrate
PEEP: Positive end-expiratory pressure
PNF: Primary nonfunction
PRBC: Packed red blood cells
ROTEM: Rotational thromboelastometry
TEG®: Thromboelastography
TOE: Transesophageal echocardiography
TRALI: Transfusion-related acute lung injury
VAE: Venous air embolism
VETs: Viscoelastic tests
